# Exercise Promotes Axon Regeneration of Newborn Striatonigral and Corticonigral Projection Neurons in Rats after Ischemic Stroke

**DOI:** 10.1371/journal.pone.0080139

**Published:** 2013-11-19

**Authors:** Qiu-Wan Zhang, Xu-Xu Deng, Xiao Sun, Jin-Xiu Xu, Feng-Yan Sun

**Affiliations:** 1 Department of Neurobiology of School of Basic Medical Sciences and Institute for Stem Cell and Regenerative Medicine of Institutes for Biomedical Science of Shanghai Medical College of Fudan University, Shanghai, P. R. China; 2 State Key Laboratory of Medical Neurobiology of Fudan University, Shanghai, P. R. China; 3 Shanghai Key Laboratory of Clinical Geriatric Medicine, Shanghai, P. R. China; Florey Institute of Neuroscience & Mental Health, Australia

## Abstract

Newborn striatal neurons induced by middle cerebral artery occlusion (MCAO) can form functional projections targeting into the substantia nigra, which should be very important for the recovery of motor function. Exercise training post-stroke improves motor recovery in clinic patients and increases striatal neurogenesis in experimental animals. This study aimed to investigate the effects of exercise on axon regeneration of newborn projection neurons in adult rat brains following ischemic stroke. Rats were subjected to a transient MCAO to induce focal cerebral ischemic injury, followed by 30 minutes of exercise training daily from 5 to 28 days after MCAO. Motor function was tested using the rotarod test. We used fluorogold (FG) nigral injection to trace striatonigral and corticonigral projection neurons, and green fluorescent protein (GFP)-targeting retroviral vectors combined with FG double labeling (GFP^+^ -FG^+^) to detect newborn projection neurons. The results showed that exercise improved the recovery of motor function of rats after MCAO. Meanwhile, exercise also increased the levels of BDNF and VEGF, and reduced Nogo-A in ischemic brain. On this condition, we further found that exercise significantly increased the number of GFP^+^ -FG^+^ neurons in the striatum and frontal and parietal cortex ipsilateral to MCAO, suggesting an increase of newborn striatonigral and corticonigral projection neurons by exercise post-stroke. In addition, we found that exercise also increased NeuN^+^ and FG^+^ cells in the striatum and frontal and parietal cortex, the ischemic territory, and tyrosine hydroxylase (TH) immunopositive staining cells in the substantia nigra, a region remote from the ischemic territory. Our results provide the first evidence that exercise can effectively enhance the capacity for regeneration of newborn projection neurons in ischemic injured mammalian brains while improving motor function. Our results provide a very important cellular mechanism to illustrate the effectiveness of rehabilitative treatment post-stroke in the clinic.

## Introduction

Stroke is the leading cause of disability and the third highest cause of death in the world [1]. In the clinic, many surviving stroke patients show morphological brain damage accompanied by neuronal function deficits in the acute phase. Fortunately, some patients recover after long-term rehabilitative treatment. Therefore, many studies have focused on understanding the mechanism of rehabilitative treatment for stroke patients in recent years [2], [3]. Previous studies have demonstrated that treadmill training can significantly reduce brain infarct volume and improve neurological outcomes after focal cerebral ischemia [4]. However, the exact molecular and cellular mechanisms are still unclear.

In the past decade, many studies have demonstrated that ischemic stroke can induce neurogenesis in both neurogenic and non-neurogenic brain regions of adult rodents [5]–[7], non-human primates [8], [9] and even humans [10]–[12]. Excitingly, researchers have found that these newly generated striatal neurons can become morphologically mature neurons [13], [14] and functionally integrate into local neural networks as indicated by electron microscopy, electrophysiological recording and FM dye imaging [15], [16]. These newborn striatal neurons also possess the capacity to receive inputs and send projections into other brain regions such as the substantia nigra (SN) [17]. It is well known that normal activity of striatonigral pathways is pivotal for maintaining motor function [18], [19] and survival of DA neurons in the SN [20]. Recent researches have reported that MCAO stroke causes degeneration of nigral dopaminergic neurons in the brains following ischemic neuronal death in the striatum and cerebral cortex, ischemic core [21], [22]. Therefore, promotion of regeneration of newborn striatonigral projections is fundamentally important for the recovery of motor function in mammalian brains after ischemic injury.

Exercise can enhance neurogenesis in the dentate gyrus of normal [23] and ischemic injured animals [24] via promoting proliferation of neural progenitors and survival of newborn neurons [25]. Traumatic and ischemic brain injury increases the level of Nogo-A, an axon growth inhibitor, and reduces synaptophysin, a synaptic protein, in adult rat brains [26], [27], and exercise can counteract the effects of traumatic brain injury on Nogo-A and synaptophysin [26]. Therefore, we asked if exercise post-stroke could improve the axon-regenerative capacity of newborn neurons in the ischemic brain.

In the present study, we used a transient MCAO to induce focal cerebral ischemia in rats, followed by GFP-gene-bearing retrovirus ventricle injection combined with FG nigral injection to trace newborn projection neurons in the striatum and cerebral cortex. Treadmill training was given to rats after MCAO. Double immunostaining and rotarod test were used to investigate the effects of exercise on newborn projection neurons and motor function. We found that the treadmill training in rats with ischemic stroke significantly increased the numbers of newborn striatonigral and corticonigral projection neurons and improved motor function recovery. Additionally, we found that exercise post-stoke protected nigral dopaminergic neurons against ischemic death. Our results suggest that rehabilitative therapy after stroke is beneficial for regeneration of neural circuitry between the nuclei in injured brains, thereby improving neural functional repair.

## Materials and Methods

### Ethics Statement

Adult male Sprague–Dawley rats with a body weight of 250 to 280 g (n = 25) from Shanghai Experimental Animal Center of Chinese Academy of Sciences were used in our experiments. The protocol was approved by the Ethics Committees of Experimental Research of Shanghai Medical College of Fudan University (Permit Number: 2011-128). This study was conducted in accordance with the National Institutes of Health Guide for the Care and Use of Laboratory Animals. All efforts were made to minimize animal suffering and reduce the number of animals used.

### Transient Middle Cerebral Artery Occlusion

Rats were anesthetized with 10% chloral hydrate (360 mg/kg, i.p.) and Arterial blood gases (pO_2_ and pCO_2_) and pH were measured with an AVL 990 Blood Gas Analyzer (AVL Co., Graz, Austria). The rats with normal blood gases were subjected to left MCAO for 30 minutes as described in detail previously [28]. During the process of ischemia, rectal temperature was monitored and maintained at 37±0.5°C with a heat lamp. Cerebral blood flow (CBF) was detected with a Laser Doppler perfusion monitor (PF5010, Perimed AB, Jarfalla, Sweden) and rats of which CBF dropped to <20% of the baseline were used for further research.

### Treadmill Training

Prior to receiving the MCAO operation, all rats were given 10 minutes treadmill pre-training twice daily for 3 days at a speed of 5.5 m/min with an electric motor-driven treadmill machine (Five-lane treadmill, JWFU, Shanghai, China) as previously described [29]. Rats were randomly divided into MCAO (n = 12) and MCAO+Exercise (MCAO+Ex) groups (n = 13). Rats in MCAO+Ex group were further subjected to 30 minutes training daily at a speed of 12 m/min from 5 to 28 days (5 days per week) after MCAO operation. Rats in MCAO group were given identical handled treatment except for the omission of forced running-speed post-stroke.

### Retrovirus and Fluorogold Injections

To label the proliferating cells in ischemic brains, pFB-hrGFP (humanized recombinant green fluorescent protein, hrGFP) retroviral supernatant (Stratagene, USA) was stereotaxically delivered into the contralateral ventricle (AP, 0.8 mm; ML, 1.4 mm; DV, 3.6 mm) at the volume of 3 µl containing 2×10^6^ infectious units on day 1 before MCAO [17]. To trace projection neurons, rats received a stereotaxic injection of fluorogold (FG; total volume: 0.3 µl of 2% FG solution in 0.9% saline; Biotium Company, CA, USA) into the ipsilateral substantia nigra (AP 5.2 mm, ML 2.5 mm, DV 8.0 mm) at 12 weeks after MCAO as described previously [17], [30]. FG nigral injection was identified in each rat using a fluorescent microscope (DM IRB, Leica, Germany) at 7 days after FG injection. Only the rats with exact nigral injection identified on brain coronal sections were selected for further investigation.

### Neurological Score Test

The neurological score of each rat was evaluated at 1, 3, 7 days post-MCAO according to Longa's scale [31]. Rats were assigned a score between 0 and 4: 0, normal motor function; 1, flexion of torso and the contralateral forelimb upon lifting of the animal by the tail; 2, circling to the contralateral side, but normal posture at rest; 3, leaning to contralateral side at rest; 4, no spontaneous motor activity.

### Motor Behavioral Test

The rotarod test was used to test motor function of rats pre-MCAO and at 3, 7, 14, 21, 28, 35, 42, 49, 56 and 63 days post-MCAO. Rats were placed on a rotarod (ENV-575MA, Med-Associates, Georgia, USA) accelerating from 4 to 40 rpm over a 5-minute period, with three repeated trials in an one-minute interval time. The average of the three recorded times for which the rat stayed on the rotarod was used to indicate motor function based on previously reported [32].

### Western Blotting

For immunoblotting analysis with various antibodies, the protein lysates from the ipsilateral fresh striatum and cerebral cortex tissues were prepared and separated on either 8% or 12% SDS-polyacrylamide gels, and transferred onto polyvinyldifluoridine (PVDF) membranes (Bio-Rad). The membranes were then blocked with 10% non-fat milk in Tris-HCl (10 mM, pH 7.4) containing 150 mM NaCl, and 1% Nonidet P-40, and separately incubated with the following specific antibodies at 4°C overnight: rabbit polyclonal anti-brain-derived neurotrophic factor (BDNF, Santa Cruz Biotechnology, Santa Cruz, CA, USA,1∶500 dilution), rabbit polyclonal anti-synapsin (SYN, Sigma, USA, 1∶1000), rabbit monoclonal anti-growth associated protein 43 (GAP43, Chemicon, Temecula, CA, USA, 1∶1000), mouse polyclonal anti-neurite growth inhibitor-A (Nogo-A, BD Transduction Laboratories, 1∶1000), rabbit polyclonal anti-vascular endothelial growth factor (VEGF, Santa Cruz 1∶500), goat polyclonal anti-glial fibrillary acidic protein (GFAP, Abcam, Cambridge, UK, 1∶2000), mouse monoclonal anti-tyrosine hydroxylase (TH, Sigma, 1∶1000), or mouse monoclonal anti-actin (Sigma, 1∶1000). After washing, the membranes were incubated with horseradish peroxidase-conjugated goat anti-rabbit IgG (1∶3000), anti-mouse IgG (1∶3000) or donkey anti-goat IgG (1∶3000) accordingly. The immunoreactivity was visualized by ECL. Relative intensities of the protein bands were quantified by scanning densitometry using image software (Image J software, National Institutes of Health, USA). Actin levels were used as internal standards.

### Immunohistochemical Staining

Rats were deeply anesthetized, and perfused intracardially with 0.9% saline solution, and 4% paraformaldehyde (PA) dissolved in 0.1 M phosphate-buffer (pH 7.4). Brains were removed, postfixed for 6 h and immersed in 20% and 30% sucrose solution until sinking [15]. Coronal brain sections were cut at a thickness of 30 µm at the Bregma level from 1.60 mm to −6.30 mm using a freezing microtome (Jung Histocut, Model 820-II, Leica, Germany) and stored at −20°C in a cryoprotectant solution for histological analysis.

For single immunostaining, sections were incubated with antibodies against mouse monoclonal anti-NeuN (1∶1000, Abcam, Cambridge, UK), rabbit polyclonal anti-FG (Chemicon, Temecula, CA, USA, 1∶4000) or mouse monoclonal anti-TH (1∶1000) overnight at 4°C. The sections treated with FG and TH antibodies were incubated with corresponding biotinylated secondary antibodies (Vector Laboratories, 1∶200) followed by avidin–biotin–peroxidase (1∶200), and the immunoreactivity was visualized with 0.05% diaminobenzidine (DAB, Sigma). The sections treated with NeuN antibody were incubated with biotinylated anti-mouse IgG (1∶200) and avidin–biotin–alkaline phosphatase complex (1∶200) and the immunoreactivity was revealed with Vector Blue. Negative controls received identical treatment except for the omission of primary antibodies and showed no specific staining.

For double staining of GFP and FG, the sections were incubated with rabbit polyclonal anti-FG antibody (1∶4000) as described above single staining, followed by biotinylated anti-rabbit IgG (1∶200), then with avidin–biotin–alkaline phosphatase complex (1∶200). FG immunoreactivity (FG^+^) was visualized with Vector Blue. After washing, the sections were incubated with a mouse monoclonal anti-GFP antibody (1∶1000), then with corresponding biotinylated secondary antibody followed by avidin–biotin–peroxidase complex (1∶200). Immunoreactivity was visualized by 0.05% DAB. Negative controls received the same process except for the omission of primary antibodies and showed no specific staining.

### Fluorescence Immunolabeling and Confocal Microscopy

FG fluorescence can be directly detected at excitation 380 nm and emission 530 nm under fluorescent microscope. Therefore, to detect triple fluorescent signals of GFP, NeuN and FG, we did double immunofluorescent staining for GFP and NeuN. The sections were simultaneously incubated with antibodies against goat polyclonal anti-GFP (1∶1000, Abcam, Cambridge, UK) and mouse monoclonal anti-NeuN (1∶1000) at 4°C overnight. The signals of GFP and NeuN immunoreactivity were revealed by anti-goat IgG-FITC (1∶500) and anti-mouse IgG-rhodamine (1∶50) secondary antibodies, respectively. After washing, the sections were mounted on glass slides and coverslipped using fluorescence mounting media. The triple fluorescent signals were detected at excitation 535 nm and emission 565 nm (rhodamine), 488 nm and 525 nm (FITC), 380 nm and 530 nm (FG) by confocal laser scanning microscopy (TCS SP5, Leica, Germany).

### Data Quantification and Statistical Analysis

The person who did functional test and cell counting were blinded during the experiments.

Sections underwent the same fixation, sectioning, and immunostaining procedures described above in preparation for unbiased stereological cell counting using the optical fractionator method assisted with Stereo Investigator 6.5 software (Micro Bright Field Inc., Williston, VT, USA). Four coronal sections (30 µm, every 12th section between 1.0 and −0.20 mm from the Bregma) of each rat brain following NeuN, GFP, FG and double GFP-FG immunostaining were taken for striatal and cortical stereological quantification. Four coronal sections (30 µm, every 10th section between −4.80 and −5.80 mm from the Bregma) of each brain were immunostained for TH and subjected to substantia nigral stereological quantification as previously described [33]. The counting criteria included the presence of signal protein immunoreactivity in the cells with a clearly neuronal phenotype.

Areas of interest were outlined at 5× magnification for volume estimation. Counting was performed with a 20× object lens of light microscopy (Q570IW, Leica, Germany) in which the counting frame size was set at 200 µm×200 µm in the striatum and cortex or 50 µm×50 µm in the substantia nigra and a height of 10 µm. Ten counting sites per section randomly chosen by software were used for cell counting. The numbers of immune-positive cells were counted in the areas as follows: TH^+^ cells in the SN, NeuN^+^, FG^+^, FG^+^-GFP^+^, GFP^+^ cells in the striatum and frontal and parietal cortex, respectively. Total volumes of each region for cell counting were automatically calculated by a Computerized Stereo Investigator 6.5 software, and the numbers of immune-positive cells were presented as cells/mm^3^ in each rat brain.

Rotarod testing data and neuro-scores were analyzed with two-way analysis of variance (ANOVA) test with least significant difference (LSD) test, followed by Student-Newman-Keuls test. Histological and Western blotting data were statistically analyzed by Unpaired Student *t*-tests and Mann-Whitney U-tests, respectively. Linear regression analysis was performed to identify correlation between Nogo-A and SYN protein levels. The data were expressed as mean ± S.E.M. Differences between groups were considered significant when *P*<0.05.

## Results

### Exercise Improved Brain Repair and Motor Function Recovery in Rats after Transient Cerebral Ischemia

**Figure 1:**
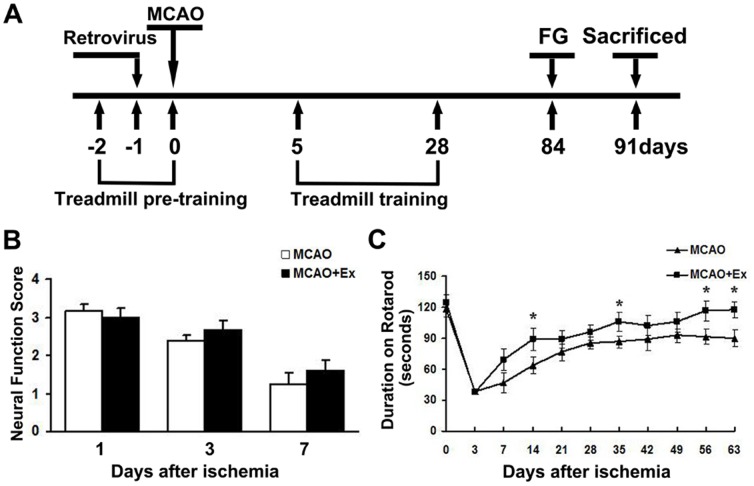
Design of animal experiments and neural function tests. A: Animals were injected with GFP-labeled retrovirus 1 day before receiving MCAO, FG injection at 84 days and sacrificed at 91 days. All animals were given treadmill exercise before MCAO and the rats in MCAO+Ex group were given treadmill exercise after MCAO. B: Neuro-score was tested at 1, 3, 7 days after MCAO. C: Motor function was assessed using the rotarod test at different time points after MCAO as shown in C. The group which received exercise post-stroke expressed better behavioral performance in rotarod test. Data are shown as mean ± S.E.M. MCAO group (n = 8); MCAO+Ex group (n = 6). *P<0.05 *vs* MCAO group.

First, we investigated whether our exercise protocols (Fig. 1A) would have long-term effects on neural repair in the brain after ischemic injury. All rats were subjected to treadmill pre-training for 3 days before MCAO and the rats in MCAO+Ex group were given treadmill training daily from 5 to 28 days after MCAO. Both MCAO+Ex and MCAO rats were subjected to rotarod tests on 3 and 7 days after the operation, and every other 7 days thereafter to evaluate motor function. Besides, neurological score were also measured at 1, 3 and 7 days post-MCAO. The results showed impairment of neurological and motor function in both MCAO+Ex and MCAO rats at the first 7 days after MCAO (Fig. 1B and C). However, MCAO+Ex rats remained on the rotating-rod longer than MCAO rats, even on 56 and 63 days after ischemic injury (Fig. 1C, *P*<0.05).

**Figure 2:**
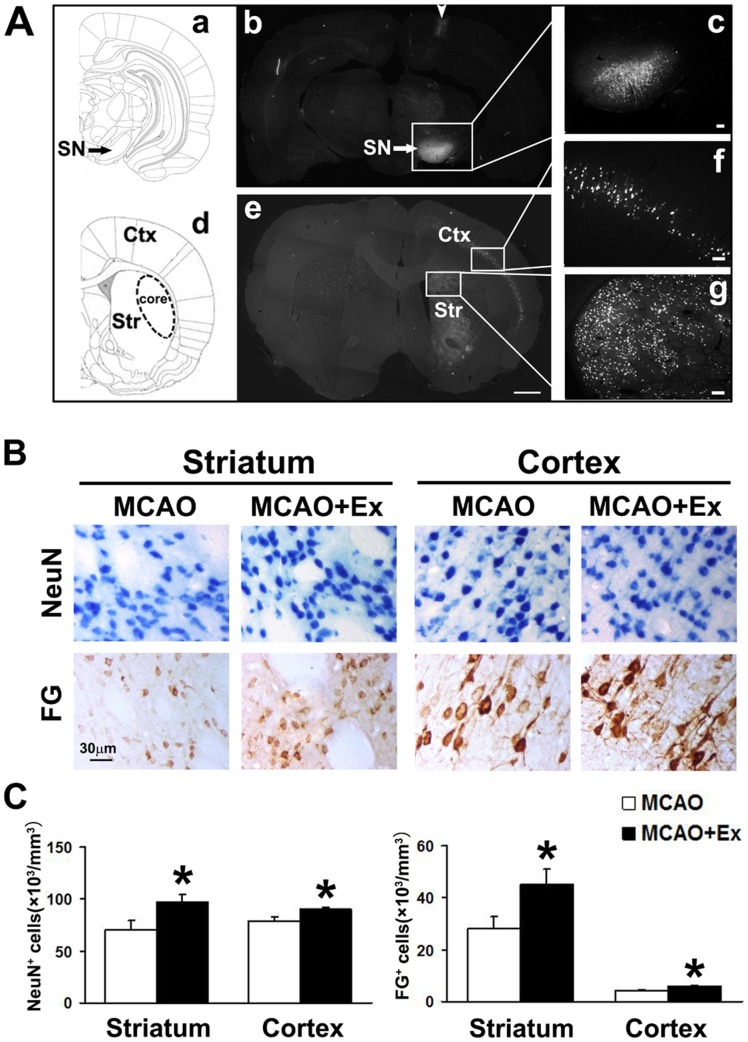
The fluorescent signal of FG and immunohistochemical signals of NeuN and FG in ischemic brain. A: Photographs showed that FG was injected into the ipsilateral SN (a, b and c) and its signals were observed in the pyramidal shaped neurons in the ipsilateral frontal and parietal cortex (f) and striatum (g) at 7 days after injection. Photographs c, f and g were magnifications of the square in photos b and e. Arrowhead in photograph b indicated where the needle was inserted toward the SN. B: Histochemical photographs displayed NeuN^+^ and FG^+^ single-labeling cells in the striatum (left) and cortex (right) of ischemic rat brain at 13 weeks after MCAO. C: Quantification showed that exercise post-stroke increased the number of NeuN^+^ and FG^+^ cells in ischemic rat brain. Data are shown as mean ± S.E.M. MCAO group (n = 8); MCAO+Ex group (n = 6). *P<0.05 *vs* MCAO group. Scale bars are 1 mm in b and e; 100 µm in c, f and g; and 30 µm in B.

With this model, we further detected that NeuN^+^ cells were significantly increased in the striatum and the frontal and parietal cortex ipsilateral to ischemia (Fig. 2 B and C, *P*<0.05) in the MCAO+Ex group compared with that in the MCAO group. To observe corticonigral and striatonigral projection neurons, we injected FG, a retrograde tracer, into the SN (Fig. 2Aa–c). Based on our present knowledge, the projection neurons in the cortex typically have pyramidal shaped cell bodies and they mainly located in the layers III, V and VI of the cortex [34]. In the current study, FG fluorescent (FG^+^) signals could be detected in striatal neurons and cortical neurons with pyramidal shape within the frontal and parietal cortex ipsilateral to nigral injection. (Fig. 2Ad–g), which was consisted with previous reported [17], [35], [36]. More interestingly,we found that the cortical and striatal FG^+^ cells were significantly increased in the MCAO+Ex rats (Fig. 2B and C, *P* *<0.05*) compared with the MCAO rats. The results indicated that exercise post-stroke could enhance the number of corticonigral and striatonigral projection neurons and improve the recovery of motor function in rats after ischemic injury induced by a transient middle cerebral artery occlusion.

### Exercise Improved Axon-regeneration of Newborn Corticonigral and Striatonigral Projection Neurons in Adult Rat Brains following a Transient Focal Ischemic Injury

**Figure 3:**
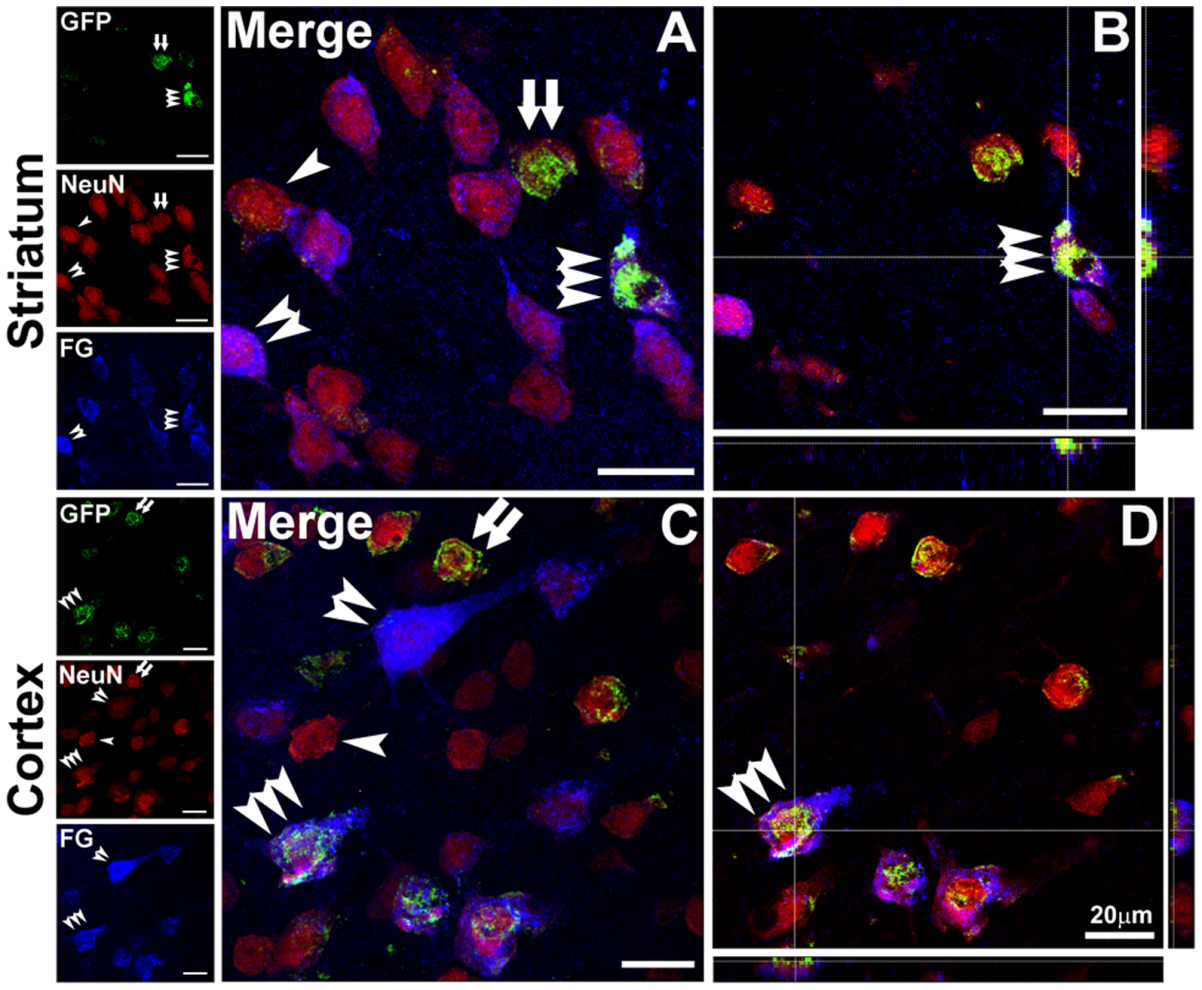
Newborn neurons formed projections to the substantia nigra in ischemic rat brain. A–D: Confocal microphotographs showed GFP^+^-NeuN^+^-FG^+^ triple-labeling cells (triple arrowheads) in the ipsilateral striatum (A and B) and parietal cortex (C and D) at 13 weeks after MCAO. NeuN^+^ single-labeling cells, GFP^+^-NeuN^+^ and NeuN^+^-FG^+^ double-labeling cells were indicated by single arrowhead, double arrows and double arrowheads, respectively. Scale bars are 20 µm.

To study whether exercise could promote the capacity to form projections of newborn neurons into their target regions in ischemic injured brains, we used FG nigral injection to trace corticonigral and striatonigral projection neurons as mentioned above, and further combined ventricle injection of GFP-targeting retroviral vectors with multiple-immunostaining to detect newborn projection neurons in the ipsilateral hemisphere to ischemia. Consistent with our previous reports [17], triple-labeled GFP^+^-NeuN^+^-FG^+^ cells were observed in ipsilateral striatum (Fig. 3A and B) at 13 weeks after MCAO. Moreover, we also detected the triple-labeled GFP^+^-NeuN^+^-FG^+^ cells in the pyramidal layer of ipsilateral cortex (Fig. 3C and D).

**Figure 4:**
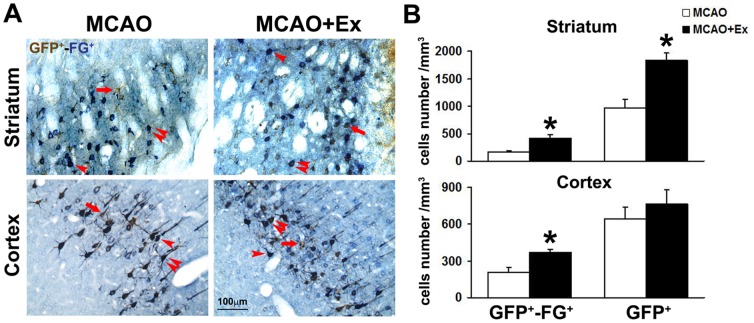
Increase of the formation of newborn projection neurons in adult ischemic brain by exercise post-stroke. Double immunohistochemical staining of FG (blue) and GFP (brown) was used to detect newly generated projection neurons. A: Histochemical photographs showed GFP^+^-FG^+^ double staining cells as indicated by double red arrowheads. GFP^+^ and FG^+^ single-labeling cells were indicated by single red arrow and arrowhead, respectively. B: Quantitative analysis showed that exercise post-stroke significantly increased the number of newborn striato- and cortico-nigral projection neurons in ischemic rat brain. Data are shown as mean ± S.E.M. MCAO group (n = 8); MCAO+Ex group (n = 6). *P<0.05 *vs* MCAO group. Scale bar in A is 100 µm.

Then, we performed double immunohistochemical staining of GFP and FG, and completed stereological counting of GFP^+^ and GFP^+^-FG^+^ cells. Figure 4A showed presence of GFP^+^-FG^+^ cells, adjacent to single GFP^+^ (brown) and FG^+^ (blue) cells, in ischemic striatum and cortex. Exercise could significantly increase the number of striatal GFP^+^ newborn cells (Fig. 4B). Moreover, the numbers of GFP^+^-FG^+^ cells in MCAO+Ex rats were increased 2.52-fold and 1.78-fold of MCAO rats in the striatum and cortex, respectively (Fig. 4B). The results suggested that exercise post-stroke could enhance capability of axon-regeneration of newborn neurons which could project into their target regions and showed their uptake and axonal retrograde transport signals from terminals into neuronal cell bodies.

### Exercise Up-regulated Expressions of Growth Factors and Down-regulated Nogo-A in Rat Brain with Ischemic Injury

**Figure 5:**
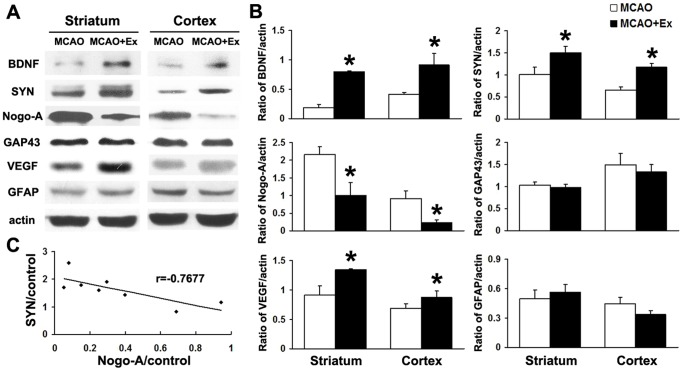
Effect of exercise on the expression of axonal growth associated protein in ischemic rat brain. Brain protein samples from rats subjected to MCAO for 30: Western blot results and corresponding densitometric analysis showed that the levels of BDNF, SYN and VEGF were increased in MCAO+Ex group compared with MCAO group; and that Nogo-A levels were reduced in the MCAO+Ex group. C: Correlation results showed that SYN levels were increased after exercise and were negatively correlated with Nogo-A levels in ischemic rat brain. Data are shown as mean ± S.E.M. *P<0.05 *vs* MCAO group; MCAO group (n = 4); MCAO+Ex group (n = 5).

We detected changes in BDNF, VEGF, GFAP, Nogo-A and synapsin expression in ischemic brains of rats using Western blot analysis. The results showed that exercise post-stroke significantly increased the expression of BDNF, VEGF and synapsin in cortical and striatal tissues. In contrast, the exercise decreased Nogo-A expression in both regions (Fig. 5A and B, *P*<0.05). Interestingly, the increase of synapsin in ischemic brains was negatively correlated with the reduction of Nogo-A (Fig. 5C).

### Exercise Increased Dopaminergic Neurons in the Substantia Nigra of Rat Brains after Ischemic Stroke

**Figure 6:**
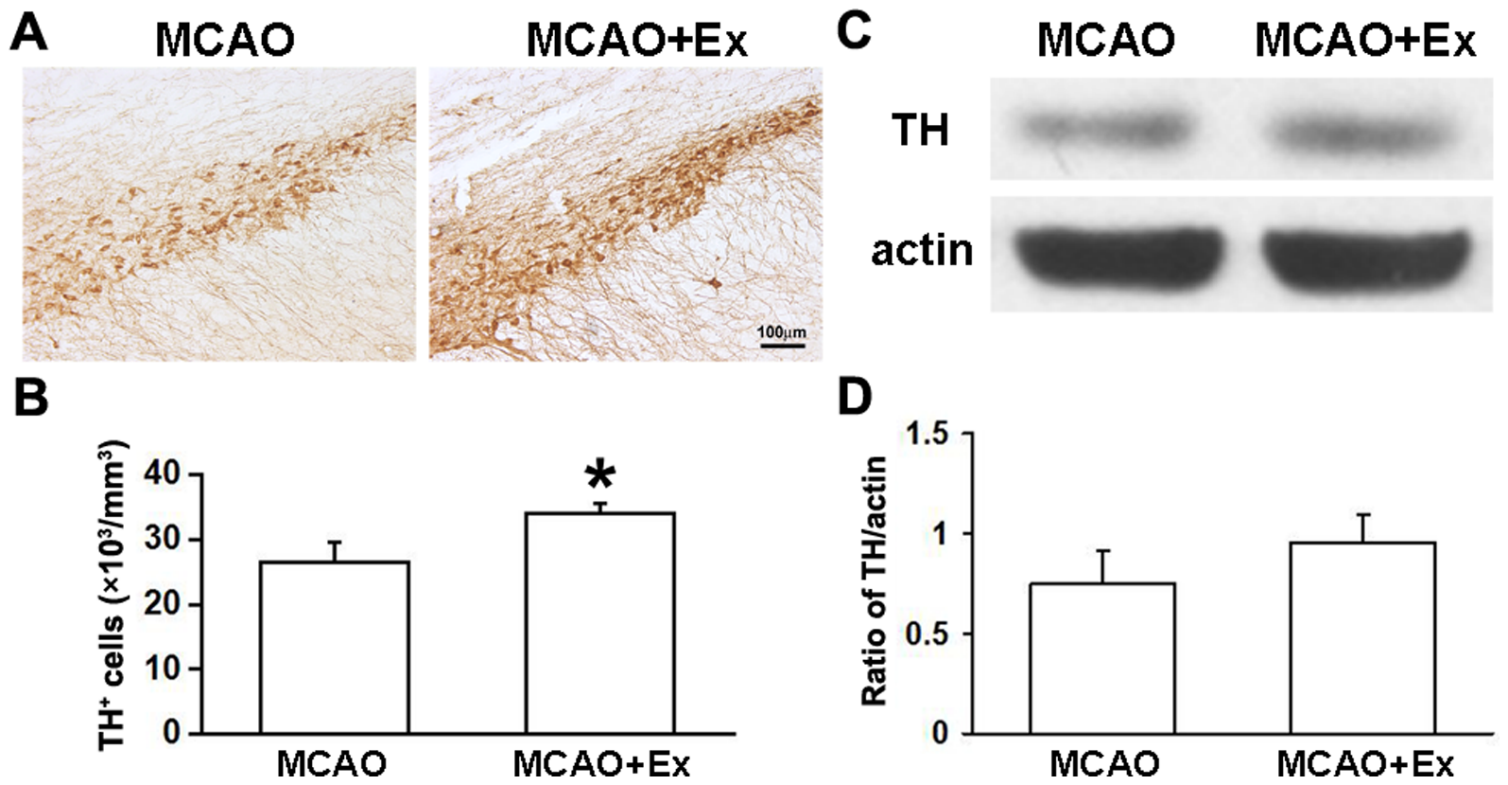
Effect of exercise on tyrosine hydroxylase expression in adult ischemic brain. A: Histochemical photographs showed TH^+^ cells in ipsilateral SN at 13 weeks after MCAO. B: Quantity analyzing data demonstrated that exercise post-stroke increased the number of TH^+^ cells in the SN. C and D: Western blot and quantitative data showed that exercise post-stroke had no significant effect on TH protein expression in the ipsilateral striatum. Data are shown as mean ± S.E.M. MCAO group (n = 4); MCAO+Ex group (n = 5). *P<0.05 *vs* MCAO group; Scale bar in A is 100 µm.

Immunostaining with TH antibody was used to detect dopamine (DA) neurons in the SN. The stereological counting results showed that a transient MCAO reduced the TH^+^ staining cells in the ipsilateral SN to the ischemia compared with that in the contralaterals (data not shown). However, exercise could reduce the loss of ipsilateral nigral dopaminergic neuron induced by a transient MCAO (Fig. 6A and B, *P*<0.05, MCAO+Ex group *vs* MCAO group). Furthermore, we detected the expressive levels of TH protein in the ipsilateral striatal tissues with Western blot analysis. The results showed that the exercise post-stroke did not significantly change the TH levels although it slightly increased (Fig. 6C and D).

## Discussion

The present study revealed that exercise post-stroke could effectively enhance the capacity for axon-regeneration of newborn striatonigral and corticonigral projection neurons in ischemic injured brains of adult rats while improving the recovery of motor behavioral function. Our results provide a very important cellular mechanism to illustrate the effectiveness of rehabilitative treatment after ischemic stroke.

BDNF and VEGF can improve neural growth and plasticity in intact [37], [38] and injured brains [39]–[41], whereas Nogo-A inhibits axonal growth [42], [43]. As mentioned before, exercise can modulate the changes of these factors in the traumatic or ischemic injured brain [26], [27]. Consistent with these reports, our results also showed that exercise significantly up-regulated BDNF expression and down-regulated Nogo-A in ischemic injured brains (Fig. 5). Moreover, the decrease in Nogo-A was negatively correlated to the increase in synapsin. In the present study, we interestingly found that exercise could up-regulate VEGF expression in the ischemic injured brain (Fig. 5). VEGF expression should be beneficial for the neuroprotection and brain repair since numerous studies have demonstrated that exogenous administration of VEGF could significantly reduce neuronal death [44] and increase neurogenesis, as well as angiogenesis in the ischemic injured brain [14], [45], [46] and modulate neural plasticity in normal brains [47]–[49]. Present study did confirm that exercise post-stroke could protect neurons against ischemic injury or increase neuron ischemic tolerance in rat brains after stroke [50]. For example, exercise post-stroke increased the number of (1) FG^+^ striatonigral and corticonigral projection neurons (Fig. 2B and C, right); (2) striatal and cortical neurons (NeuN^+^ cells, Fig. 2B and C, left); and (3) nigral DA neurons (TH^+^ cells) in the ipsilateral hemispheres to MCAO (Fig. 6A and B). Putting together, we found that exercise post-stroke enhanced endogenous neuroprotective effects, which should be associated with regulation of BDNF, VEGF and Nogo-A expression in the ischemic injured brains. Under this experimental condition, we further investigated effects of exercise post-stroke on axon/neurite development of newborn projection neurons in ischemic injured brains.

It is well known that stroke-induced neurogenesis exists in the striatum and cortex of adult rat brains and can be enhanced by several growth factors and anti-apoptotic factors [13], [14], [46], [51]. Reestablishment of neural networks of newborn neurons is the fundamental anatomic basis for functional repair in the damaged brains. In past years, it has been demonstrated that new neurons generated after ischemic injury can form synapses and locally integrate with preexisting neurons in the injured mammalian brain [15], [16]. Moreover, newborn striatal neurons can develop long axons to project into the SN, a target region, and form functional striatonigral projections [17], [30]. Present study confirmed our previous reports (Fig. 3 and 4). We further found that newly generated cortical neurons could also form projections targeting to the SN as indicated by GFP^+^-NeuN^+^-FG^+^ cells in the pyramidal layer of cerebral cortex (Fig. 3C–D), suggesting that new cortical neurons could become corticonigral projection neurons. Furthermore, these newborn corticonigral and striatonigral neurons possessed functional axon/projections capable of presynaptic uptake and retrogradely axonal transportation because FG after nigral injections could be detected in the GFP^+^ cortical and striatal neurons. Previous study has demonstrated that the axon regeneration of new neurons can be increased by exogenous overexpression of Bcl-2 in ischemic injured brains [30]. In the present study, we revealed that the axon-regeneration of newborn projection neurons could be promoted by exercise post-stroke (Fig. 4). These phenomena suggest that physical rehabilitative training post-stroke can accelerate the axon regeneration of newborn projection neurons and/or reestablishment of their axonal transporting function via activation of endogenous mechanisms.

In addition, we found that exercise post-stroke could significantly increase DA neurons in the ipsilateral SN (Fig. 6). Although we have no way to directly identify whether the increase in nigral DA neurons by exercise is the result of reorganization/reconstruction of the corticonigral and striatonigral neural pathways, present results strongly indicated that exercise post-stroke could effectively enhance restoration of functional neural circuitry within the basal ganglia. The evidences included that exercise post-stroke increased (1) the number of newly formed striatonigral and corticonigral projection neurons; (2) the uptake of newly formed axon-terminals; (3) retrogradely transportation of newly formed axon from terminals in the SN into neuronal cell body in the cortex and striatum (Fig. 4); (4) the number of nigral DA neurons (Fig. 6). Both the striatum and SN are important nuclei within the basal ganglia and play critical roles in the maintenance of motor function [52]. Theoretically, increases in the numbers of functional striatal and cortical newborn projection neurons or nigral DA neurons by exercise post-stroke should be beneficial for the recovery of motor function. Supporting this point of view, our results showed that exercise post-stroke significantly improved motor behavior in rats after ischemic stroke (Fig. 1C). Taken together, our data suggest that reconstruction of new neural networks within the nuclei have important roles in function repair of adult brain, and that exercise may be valuable for morphological and functional repairs in the injured adult mammalian brains.

In summary, present study provided the first evidence that passive exercise post-stroke could effectively improve axonal-regeneration of newborn projection neurons and accelerate the reestablishment of new neuronal circuitries within the basal ganglia after ischemic injury, which should provide very important anatomical foundation for the recovery of motor behavioral function. Our results provided cellular mechanisms of passive rehabilitative treatment following stroke in the clinic.

## References

[1] Nakayama H, Jorgensen HS, Raaschou HO, Olsen TS (1994) Recovery of upper extremity function in stroke patients: the Copenhagen Stroke Study. Arch Phys Med Rehabil 75: 394–398. 8172497 10.1016/0003-9993(94)90161-9

[2] Cho HY, Kim JS, Lee GC (2012) Effects of motor imagery training on balance and gait abilities in post-stroke patients: a randomized controlled trial. Clin Rehabil. 10.1177/0269215512464702 23129815

[3] Metrot J, Mottet D, Hauret I, van Dokkum L, Bonnin-Koang HY, et al (2012) Changes in Bimanual Coordination During the First 6 Weeks After Moderate Hemiparetic Stroke. Neurorehabil Neural Repair. 10.1177/1545968312461072 23135767

[4] Yang YR, Wang RY, Wang PS, Yu SM (2003) Treadmill training effects on neurological outcome after middle cerebral artery occlusion in rats. Can J Neurol Sci 30: 252–258. 12945951 10.1017/s0317167100002687

[5] Gage FH (2000) Mammalian neural stem cells. Science 287: 1433–1438. 10688783 10.1126/science.287.5457.1433

[6] van Praag H, Schinder AF, Christie BR, Toni N, Palmer TD, et al (2002) Functional neurogenesis in the adult hippocampus. Nature 415: 1030–1034. 11875571 10.1038/4151030a PMC9284568

[7] Lichtenwalner RJ, Parent JM (2006) Adult neurogenesis and the ischemic forebrain. J Cereb Blood Flow Metab 26: 1–20. 15959458 10.1038/sj.jcbfm.9600170

[8] Darsalia V, Heldmann U, Lindvall O, Kokaia Z (2005) Stroke-induced neurogenesis in aged brain. Stroke 36: 1790–1795. 16002766 10.1161/01.STR.0000173151.36031.be

[9] Kuge A, Takemura S, Kokubo Y, Sato S, Goto K, et al (2009) Temporal profile of neurogenesis in the subventricular zone, dentate gyrus and cerebral cortex following transient focal cerebral ischemia. Neurol Res 31: 969–976. 19138475 10.1179/174313209X383312

[10] Jin K, Wang X, Xie L, Mao XO, Zhu W, et al (2006) Evidence for stroke-induced neurogenesis in the human brain. Proc Natl Acad Sci U S A 103: 13198–13202. 16924107 10.1073/pnas.0603512103 PMC1559776

[11] Macas J, Nern C, Plate KH, Momma S (2006) Increased generation of neuronal progenitors after ischemic injury in the aged adult human forebrain. J Neurosci 26: 13114–13119. 17167100 10.1523/JNEUROSCI.4667-06.2006 PMC6674966

[12] Minger SL, Ekonomou A, Carta EM, Chinoy A, Perry RH, et al (2007) Endogenous neurogenesis in the human brain following cerebral infarction. Regen Med 2: 69–74. 17465777 10.2217/17460751.2.1.69

[13] Arvidsson A, Collin T, Kirik D, Kokaia Z, Lindvall O (2002) Neuronal replacement from endogenous precursors in the adult brain after stroke. Nat Med 8: 963–970. 12161747 10.1038/nm747

[14] Wang YQ, Guo X, Qiu MH, Feng XY, Sun FY (2007) VEGF overexpression enhances striatal neurogenesis in brain of adult rat after a transient middle cerebral artery occlusion. J Neurosci Res 85: 73–82. 17061257 10.1002/jnr.21091

[15] Hou SW, Wang YQ, Xu M, Shen DH, Wang JJ, et al (2008) Functional integration of newly generated neurons into striatum after cerebral ischemia in the adult rat brain. Stroke 39: 2837–2844. 18635857 10.1161/STROKEAHA.107.510982

[16] Yang SZ, Li KY, Wang YQ, Shen DH, Sun FY (2010) Functional contribution of neurogenesis in normal and stroke brain. In: Jin KL, editor. Adult Neurogenesis and Central Nervous System Diseases. Kerala, India: Transworld Research Network. pp. 91–125.

[17] Sun X, Zhang QW, Xu M, Guo JJ, Shen SW, et al (2012) New striatal neurons form projections to substantia nigra in adult rat brain after stroke. Neurobiol Dis 45: 601–609. 22005319 10.1016/j.nbd.2011.09.018

[18] Martin AB, Fernandez-Espejo E, Ferrer B, Gorriti MA, Bilbao A, et al (2008) Expression and function of CB1 receptor in the rat striatum: localization and effects on D1 and D2 dopamine receptor-mediated motor behaviors. Neuropsychopharmacology 33: 1667–1679. 17957223 10.1038/sj.npp.1301558

[19] Thompson LH, Grealish S, Kirik D, Bjorklund A (2009) Reconstruction of the nigrostriatal dopamine pathway in the adult mouse brain. Eur J Neurosci 30: 625–638. 19674082 10.1111/j.1460-9568.2009.06878.x

[20] Mallet N, Ballion B, Le Moine C, Gonon F (2006) Cortical inputs and GABA interneurons imbalance projection neurons in the striatum of parkinsonian rats. J Neurosci 26: 3875–3884. 16597742 10.1523/JNEUROSCI.4439-05.2006 PMC6674115

[21] Nakane M, Teraoka A, Asato R, Tamura A (1992) Degeneration of the ipsilateral substantia nigra following cerebral infarction in the striatum. Stroke 23: 328–332. 1542891 10.1161/01.str.23.3.328

[22] Tamura A, Kirino T, Sano K, Takagi K, Oka H (1990) Atrophy of the ipsilateral substantia nigra following middle cerebral artery occlusion in the rat. Brain Res 510: 154–157. 2322841 10.1016/0006-8993(90)90744-v

[23] Speisman RB, Kumar A, Rani A, Foster TC, Ormerod BK (2012) Daily exercise improves memory, stimulates hippocampal neurogenesis and modulates immune and neuroimmune cytokines in aging rats. Brain Behav Immun. 10.1016/j.bbi.2012.09.013 PMC3545095 23078985

[24] Luo CX, Jiang J, Zhou QG, Zhu XJ, Wang W, et al (2007) Voluntary exercise-induced neurogenesis in the postischemic dentate gyrus is associated with spatial memory recovery from stroke. J Neurosci Res 85: 1637–1646. 17465031 10.1002/jnr.21317

[25] Leasure JL, Grider M (2010) The effect of mild post-stroke exercise on reactive neurogenesis and recovery of somatosensation in aged rats. Exp Neurol 226: 58–67. 20696163 10.1016/j.expneurol.2010.08.003

[26] Chytrova G, Ying Z, Gomez-Pinilla F (2008) Exercise normalizes levels of MAG and Nogo-A growth inhibitors after brain trauma. Eur J Neurosci 27: 1–11. 18093178 10.1111/j.1460-9568.2007.05982.x

[27] Weinmann O, Schnell L, Ghosh A, Montani L, Wiessner C, et al (2006) Intrathecally infused antibodies against Nogo-A penetrate the CNS and downregulate the endogenous neurite growth inhibitor Nogo-A. Mol Cell Neurosci 32: 161–173. 16697217 10.1016/j.mcn.2006.03.007

[28] Yang ZJ, Bao WL, Qiu MH, Zhang LM, Lu SD, et al (2002) Role of vascular endothelial growth factor in neuronal DNA damage and repair in rat brain following a transient cerebral ischemia. J Neurosci Res 70: 140–149. 12271463 10.1002/jnr.10380

[29] Ploughman M, Windle V, MacLellan CL, White N, Dore JJ, et al (2009) Brain-derived neurotrophic factor contributes to recovery of skilled reaching after focal ischemia in rats. Stroke 40: 1490–1495. 19164786 10.1161/STROKEAHA.108.531806

[30] Guo JJ, Liu F, Sun X, Huang JJ, Xu M, et al (2012) Bcl-2 enhances the formation of newborn striatal long-projection neurons in adult rat brain after a transient ischemic stroke. Neurosci Bull 28: 669–679. 23225311 10.1007/s12264-012-1288-5 PMC5561824

[31] Longa EZ, Weinstein PR, Carlson S, Cummins R (1989) Reversible middle cerebral artery occlusion without craniectomy in rats. Stroke 20: 84–91. 2643202 10.1161/01.str.20.1.84

[32] Chen J, Sanberg PR, Li Y, Wang L, Lu M, et al (2001) Intravenous administration of human umbilical cord blood reduces behavioral deficits after stroke in rats. Stroke 32: 2682–2688. 11692034 10.1161/hs1101.098367

[33] Wild AR, Akyol E, Brothwell SL, Kimkool P, Skepper JN, et al (2013) Memantine block depends on agonist presentation at the NMDA receptor in substantia nigra pars compacta dopamine neurones. Neuropharmacology 73C: 138–146. 10.1016/j.neuropharm.2013.05.013 PMC4363981 23727219

[34] Amaral DG (2000) The Anatomical Organization of the Central Nervous System. In: Kandel ER, Schwartz JH, Jessell TM, editor. Principles of Neural Science. New York: McGraw Hill. pp. 317–336.

[35] Haines DE (1995) Neuroanatomy: An Atlas of Structures, Sections, and System. In: Wilkins W, editor. Maryland. pp. 134–203.

[36] Trushina E, Dyer RB, Badger JD 2nd, Ure D, Eide L, et al (2004) Mutant huntingtin impairs axonal trafficking in mammalian neurons in vivo and in vitro. Mol Cell Biol 24: 8195–8209. 15340079 10.1128/MCB.24.18.8195-8209.2004 PMC515048

[37] Bramham CR, Messaoudi E (2005) BDNF function in adult synaptic plasticity: the synaptic consolidation hypothesis. Prog Neurobiol 76: 99–125. 16099088 10.1016/j.pneurobio.2005.06.003

[38] Ding Q, Ying Z, Gomez-Pinilla F (2011) Exercise influences hippocampal plasticity by modulating brain-derived neurotrophic factor processing. Neuroscience 192: 773–780. 21756980 10.1016/j.neuroscience.2011.06.032 PMC3225196

[39] MacLellan CL, Keough MB, Granter-Button S, Chernenko GA, Butt S, et al (2011) A critical threshold of rehabilitation involving brain-derived neurotrophic factor is required for poststroke recovery. Neurorehabil Neural Repair 25: 740–748. 21705652 10.1177/1545968311407517

[40] Schabitz WR, Steigleder T, Cooper-Kuhn CM, Schwab S, Sommer C, et al (2007) Intravenous brain-derived neurotrophic factor enhances poststroke sensorimotor recovery and stimulates neurogenesis. Stroke 38: 2165–2172. 17510456 10.1161/STROKEAHA.106.477331

[41] Zhu JM, Zhao YY, Chen SD, Zhang WH, Lou L, et al (2011) Functional recovery after transplantation of neural stem cells modified by brain-derived neurotrophic factor in rats with cerebral ischaemia. J Int Med Res 39: 488–498. 21672352 10.1177/147323001103900216

[42] GrandPre T, Nakamura F, Vartanian T, Strittmatter SM (2000) Identification of the Nogo inhibitor of axon regeneration as a Reticulon protein. Nature 403: 439–444. 10667797 10.1038/35000226

[43] Kilic E, ElAli A, Kilic U, Guo Z, Ugur M, et al (2010) Role of Nogo-A in neuronal survival in the reperfused ischemic brain. J Cereb Blood Flow Metab 30: 969–984. 20087369 10.1038/jcbfm.2009.268 PMC2949191

[44] Qiu MH, Zhang R, Sun FY (2003) Enhancement of ischemia-induced tyrosine phosphorylation of Kv1.2 by vascular endothelial growth factor via activation of phosphatidylinositol 3-kinase. J Neurochem 87: 1509–1517. 14713306 10.1046/j.1471-4159.2003.02110.x

[45] Jin K, Zhu Y, Sun Y, Mao XO, Xie L, et al (2002) Vascular endothelial growth factor (VEGF) stimulates neurogenesis in vitro and in vivo. Proc Natl Acad Sci U S A 99: 11946–11950. 12181492 10.1073/pnas.182296499 PMC129374

[46] Wang YQ, Cui HR, Yang SZ, Sun HP, Qiu MH, et al (2009) VEGF enhance cortical newborn neurons and their neurite development in adult rat brain after cerebral ischemia. Neurochem Int 55: 629–636. 19540294 10.1016/j.neuint.2009.06.007

[47] Ma YY, Li KY, Wang JJ, Huang YL, Huang Y, et al (2009) Vascular endothelial growth factor acutely reduces calcium influx via inhibition of the Ca2+ channels in rat hippocampal neurons. J Neurosci Res 87: 393–402. 18803284 10.1002/jnr.21859

[48] Xu JY, Zheng P, Shen DH, Yang SZ, Zhang LM, et al (2003) Vascular endothelial growth factor inhibits outward delayed-rectifier potassium currents in acutely isolated hippocampal neurons. Neuroscience 118: 59–67. 12676137 10.1016/s0306-4522(02)00948-x

[49] Sun FY, Guo X (2005) Molecular and cellular mechanisms of neuroprotection by vascular endothelial growth factor. J Neurosci Res 79: 180–184. 15573409 10.1002/jnr.20321

[50] Zhang F, Wu Y, Jia J (2011) Exercise preconditioning and brain ischemic tolerance. Neuroscience 177: 170–176. 21241780 10.1016/j.neuroscience.2011.01.018

[51] Zhang R, Xue YY, Lu SD, Wang Y, Zhang LM, et al (2006) Bcl-2 enhances neurogenesis and inhibits apoptosis of newborn neurons in adult rat brain following a transient middle cerebral artery occlusion. Neurobiol Dis 24: 345–356. 16996745 10.1016/j.nbd.2006.07.012

[52] Mink JW (1999) Basal ganglia. In: Zigmond MJ, Bloom FE, Landis SC,Roberts JL, Squire LR, editor. Fundamental Neuroscience. California: Academic Press. pp. 951–972.

